# Facilitators and Barriers to Dementia Assessment and Diagnosis: Perspectives From Dementia Experts Within a Global Health Context

**DOI:** 10.3389/fneur.2022.769360

**Published:** 2022-03-28

**Authors:** Alissa Bernstein Sideman, Tala Al-Rousan, Elena Tsoy, Stefanie D. Piña Escudero, Maritza Pintado-Caipa, Suchanan Kanjanapong, Lingani Mbakile-Mahlanza, Maira Okada de Oliveira, Myriam De la Cruz-Puebla, Stelios Zygouris, Aya Ashour Mohamed, Hany Ibrahim, Collette A. Goode, Bruce L. Miller, Victor Valcour, Katherine L. Possin

**Affiliations:** ^1^Philip R. Lee Institute for Health Policy Studies, University of California, San Francisco, San Francisco, CA, United States; ^2^Global Brain Health Institute, University of California, San Francisco, San Francisco, CA, United States; ^3^Department of Humanities and Social Sciences, University of California, San Francisco, San Francisco, CA, United States; ^4^Herbert Wertheim School of Public Health, University of California, La Jolla, CA, United States; ^5^Department of Neurology, Memory and Aging Center, University of California, San Francisco, San Francisco, CA, United States; ^6^Research Department, Peruvian Institute of Neurosciences, Lima, Peru; ^7^Division of Geriatrics, Department of Preventive Medicine, Faculty of Medicine Siriraj Hospital, Mahidol University, Bangkok, Thailand; ^8^Department of Psychology, Faculty of Social Sciences, University of Botswana, Gaborone, Botswana; ^9^Cognitive and Behavioral Neurology Unit, Hospital das Clinicas, University of São Paulo, São Paulo, Brazil; ^10^Hospital Santa Marcelina, São Paulo, São Paulo, Brazil; ^11^Global Brain Health Institute, Trinity College Dublin, Dublin, Ireland; ^12^Neurosciences Institute, Autonomous University of Barcelona, Barcelona, Spain; ^13^Cognition and Brain Plasticity Unit, University of Barcelona, Barcelona, Spain; ^14^Bellvitge Institute for Biomedical Research, Barcelona, Spain; ^15^Technical University of Ambato, Tungurahua, Ecuador; ^16^Centre for Research and Technology Hellas/Information Technologies Institute, Thessaloniki, Greece; ^17^Department of Neurology, Ain Shams University, Cairo, Egypt; ^18^Geriatric Medicine Department, Ain Shams University, Cairo, Egypt

**Keywords:** dementia, global health, dementia experts, qualitative study, cognitive assessment

## Abstract

**Objectives:**

Dementia poses one of the greatest global health challenges, affecting 50 million people worldwide. With 10 million new cases each year, dementia is a growing burden, particularly in low- and middle-income countries (LMIC). This study aimed to identify the facilitators and barriers to providing quality dementia assessment and care in LMICs from a global health perspective.

**Methods/Design:**

A qualitative semi-structured interview study with 20 dementia expert healthcare providers from 19 countries. To be included, providers had to: practice dementia assessment or care in LMICs where the population over age 60 is projected to more than double by 2050 and be recognized as a leading dementia expert in the region based on position, research publications, and/or policy leadership. Interviews were analyzed by a multidisciplinary team of researchers using thematic analysis.

**Results:**

Barriers to dementia assessment and care included stigma about dementia, poor patient engagement in and access to healthcare, inadequate linguistic and cultural validation, limited dementia capable workforce, competing healthcare system priorities, and insufficient health financing. Facilitators included the rise in dementia awareness campaigns, dementia training for general practitioners, availability of family support and family caregivers, and national and international collaborations including coordinated policy efforts and involvement in international research initiatives.

**Conclusions:**

Findings from this study provide insights for prioritizing dementia assessment and care capacity-building in LMICs as a global health priority and for tailored public health approaches to strengthen dementia assessment and care at the individual, community, national, and multi-national levels.

## Key Points

- This study used qualitative semi-structured interviews to understand and characterize the assessment and care of dementia in low- and middle-income countries from the perspective of expert healthcare providers and leaders in dementia research and policy.
- We identified four major thematic domains, and within these domains characterized facilitators and barriers based on a social-ecological model of health.
- While much of the literature focuses on dementia care in high-income countries, these findings demonstrate the challenges and strengths that exist to dementia assessment and care from the perspective of dementia experts from a broader global view.
- Based on these findings, we provide recommendations using a social-ecological model for taking a tailored public health approach to strengthening dementia care at the individual, community, national, and policy levels.

## Introduction

Dementia is a costly disease impacting patients, families, and societies, and is a major and growing global health challenge due to population aging. In 2015, Alzheimer's Disease International (ADI) reported that there were over 9.9 million new cases of dementia each year worldwide, implying one new case every 3.2 seconds (1). Of these cases, 58% of lived in LMIC, and this is expected to rise to 68% in 2050 due to changing demographics and healthcare infrastructure (1, 2). These countries are expected to face major challenges to ensure that their health and social systems are prepared to meet the increasing dementia care needs associated with this demographic shift.

Research has demonstrated that people with dementia and their caregivers are not receiving services of the type and quality that they need and there are wide variations in detecting dementia globally (3). Pragmatic, realistic strategies and policies that address key facilitators and barriers to quality dementia care are crucially needed (4, 5). Within the countries experiencing the most rapid growth in the aging population, dementia care experts who are providing clinical services on the ground as well as leading research and policy initiatives could provide a critical perspective to inform the development of National Dementia Plans and brain health diplomacy initiatives (6).

The purpose of this study was to identify facilitators and barriers to meeting dementia care needs that are common across countries experiencing a rapid increase in the aging population. Our approach was to collect insights from in-depth interviews with leading regional dementia experts who are working to address gaps in dementia care through their clinical work, research, and/or policy work. Knowledge gained in these expert interviews may offer insight for developing national and international strategies and interventions.

### Social-Ecological Model

The social-ecological model is a framework that suggests people's experiences are shaped by interactions among individual, interpersonal, institutional, community, and policy factors (7). Following analysis of our data, we identified the social-ecological model as an approach to organizing our findings, as it can be used to provide a way of understanding how both social and structural factors shape dementia assessment and care approaches in global health settings. A social-ecological model has been used in prior studies to understand attitudes about dementia, access to dementia care, and dementia awareness (8–10). The model is dynamic in that some findings may fall into more than one category. We demonstrate ways components of the model can collectively guide efforts to improve dementia care globally by either developing new interventions or building on strengths and approaches that already exist.

## Methods

### Design

We conducted a qualitative study based on interviews with dementia experts from different regions of the world, focused primarily on those in LMICs. Interviewers from our multidisciplinary team had expertise in global health, social science, neurology, and neuropsychology. Interviewers were trained as part of their Global Brain Health Institute fellowship by the first author, a medical anthropologist with 15 years of qualitative research experience. The study was conducted in accordance with the Consolidated Criteria for Reporting Qualitative Research (COREQ) reporting guidelines (Supplementary Materials, Appendix 1).

### Participants and Setting

We selected experts in dementia diagnosis and care using purposeful sampling (11), which involved identifying and interviewing individuals who are knowledgeable about dementia. Inclusion criteria were: (1) dementia experts in countries with projected growth of the population aged 60 years or older of over 100% between 2017 and 2050 based on United Nations Department of Economic and Social Affairs Population Division estimates (12); (2) established expertise in dementia diagnosis and care, including the following disciplines: geriatric medicine, geriatric psychiatry, neurology, neuropsychology, and psychiatry; (3) currently involved in clinical practice and training; and (4) recognition as a leading national dementia expert based on role, publications, and/or policy leadership. We first identified a list of possible participants based on recommendations from professional networks, local Alzheimer's Associations, and multi-national studies. We then contacted experts by email to invite them to participate in the study. We contacted a total of 26 experts and 20 responded and agreed to participate. We conducted interviews from August 2019 through November 2020. All participants provided oral informed consent. This study was approved by the Institutional Review Board at the University of California, San Francisco.

### Data Collection

Our multidisciplinary team developed a pre-interview survey (Supplementary materials, Appendix 2) and a semi-structured interview guide (Supplementary materials, Appendix 3). Study instruments were reviewed by the team, and then piloted with a neurologist. The interview covered the following domains: (1) expert and national context; (2) process for diagnosing dementia; (3) facilitators and barriers to dementia diagnosis and care; (4) needs to improve dementia diagnosis and care. While the interview guide included structured questions, the interviews were open-ended, meaning that interviewers were able to ask follow-up questions, request examples, and expand on topics as long as all key domains of the interview guide were covered. Once an expert was identified, we sent the survey *via* a password-protected online platform and scheduled an interview. Interviews were conducted in English or the expert's native language, based on their preference, by trained researchers (AS, ET, SE, MP-C, SK, TA-R, LM-M, MdO, MlCP, SZ, AM, and HI) *via* secure videoconferencing, and lasted approximately 1 h. They were digitally recorded and transcribed. If the interview was not conducted in English, it was translated for analysis.

### Data Analysis

We used inductive and deductive thematic analysis to analyze the data and identify key themes through an iterative process using ATLAS.ti. A team of 3–5 coders inductively coded 5/20 transcripts, meeting regularly to review the codes. We discussed and resolved discrepancies in coding and developed a codebook based on consensus. The codebook was reviewed by the first, second, and senior authors. The first author then used this codebook to code the remaining transcripts (15/20). When new codes emerged, they were reviewed by the team during regular meetings and added to the codebook. Disagreements were discussed and resolved. We reached thematic saturation when no new codes emerged, though we continued to code all transcripts. The team then identified themes based on the codes and themes were deductively organized based on the social-ecological framework, our overarching theoretical model. We reviewed the data for each theme and identified the most illustrative quotations to incorporate into the manuscript and tables.

## Results

### Participants

We provide characteristics of the 19 countries represented by our 20 research participants in Table 1 and demographic and practice characteristics of our sample in Table 2. Ten participants were neurologists, four were neuropsychologists, three were geriatricians, two were psychiatrists, and one was a geriatric psychiatrist.

**Table 1:** Characteristics of the 19 countries represented by experts in the study^a^.

**World region**	**Country**	**Projected growth (%) (2)^b^**	**Literacy rate (%) (13)^c^**	**Mean years of school (13)^d^**	**Rural residence (%) (13)^e^**	**GDPPC (USD) (13)^e^**	**Gini index (14)^f^**	**Linguistic diversity index (15)^g^**
Africa	Botswana	264	40	ND	31	$8,259	53.3	0.444
Africa	Kenya	247	57	ND	73	$1,711	40.8	0.901
Africa	Ghana	185	51	ND	44	$2,202	43.5	0.805
Americas	Brazil	235	79	8.0	13	$8,921	53.9	0.032
Americas	Colombia	237	83	8.5	19	$6,651	50.4	0.030
Americas	Ecuador	208	73	8.8	36	$6,345	45.4	0.264
Americas	Mexico	244	81	8.9	20	$9,698	45.4	0.135
Americas	Nicaragua	276	56	ND	41	$2,029	46.2	0.081
Americas	Peru	219	79	9.7	22	$6,947	42.8	0.376
Asia	India	203	45	ND	66	$2,016	37.8	0.930
Asia	Indonesia	223	74	8.2	45	$3,894	39.0	0.846
Asia	Kyrgyzstan	220	97	ND	64	$1,281	27.7	0.670
Asia	Taiwan	204	ND	ND	ND	ND	ND	ND
Asia	Thailand	208	79	8.5	50	$7,274	36.4	0.753
Europe	Greece	157	95	10.3	21	$20,324	34.4	0.175
Europe	Spain	166	95	10.3	20	$30,524	34.7	0.438
Middle East	Egypt	195	33	9.0	57	$2,549	31.5	0.509
Middle East	Jordan	270	91	ND	9	$4,248	33.7	0.484
Middle East	UAE	779	69	12.5	13	$43,005	32.5	0.777

*GDPPC, gross domestic product per capita; ND, no data; UAE, United Arab Emirates*.

a *Adapted from Tsoy et al. (16)*.

b *Projected growth of population aged 60 and above between 2017 and 2050*.

c *Literacy rate among population aged 65 and above; reference year: 2013 (Botswana), 2015 (Nicaragua, Thailand, UAE), 2017 (Ecuador, Egypt), 2018 (Brazil, Colombia, Greece, India, Indonesia, Jordan, Kenya, Kyrgyzstan, Mexico, Peru, Spain)*.

d *Mean years of formal schooling among adults aged 25 and above; reference year: 2016 (Greece), 2017 (Ecuador, Egypt), 2018 (Brazil, Colombia, Indonesia, Mexico, Peru, Spain, Thailand, UAE)*.

e * Rural residence among adults aged 25 and above; reference year: 2018 (all)*.

f *Gini index measures the deviation of the actual income distribution from a hypothetical perfectly equal distribution with values ranging from 0 (perfect equality) to 100 (perfect inequality); reference year: 2010 (Jordan), 2011 (India), 2014 (Nicaragua, UAE), 2015 (Botswana, Kenya), 2017 (Egypt, Greece, Spain), 2018 (Brazil, Colombia, Ecuador, Indonesia, Kyrgyzstan, Mexico, Peru, Thailand)*.

g *Linguistic diversity index is based on the population of each language spoken in the country as a proportion of the total population with values ranging from 0 (no diversity, everyone has the same primary language) to 1 (total diversity, no two people have the same primary language); reference year: 2009 (all)*.

**Table 2:** Participant demographics and practice characteristics.

**Region**	***n***
**Demographic information (*****n*** **=** **20)**	
Africa	4
Asia	6
Europe	2
Central/South America	6
Middle East	2
**Gender**	
Female	9
Male	11
**Primary specialty**	
Geriatric medicine	3
Geriatric psychiatry	1
Neurology	10
Neuropsychology	4
Psychiatry	2
**Years in clinical practice**	***Mean (SD)***
	21.3 (10.8)
Practice Characteristics^*^	
Expert Practice Setting^**^	***n***
Public institution	8
Private institution	9
Teaching hospital	13
Research institution or university	8
Day Care Center	1
**% time dedicated to the following activities**	***Mean % (SD)***
Patient care	39 (20)
Research	25 (18)
Teaching & mentoring	22 (14)
Administration	11 (9)
Other	2 (7)
**Number of patients newly diagnosed with MCI or Dementia per month at expert's practice**	***Mean (SD)***
	25 (19)

* *One provider did not provide responses to all non-demographic questions*.

** *Some providers indicated working in multiple practice settings*.

### Barriers and Facilitators According to a Social Ecological Model Framework

**Figure 1:**
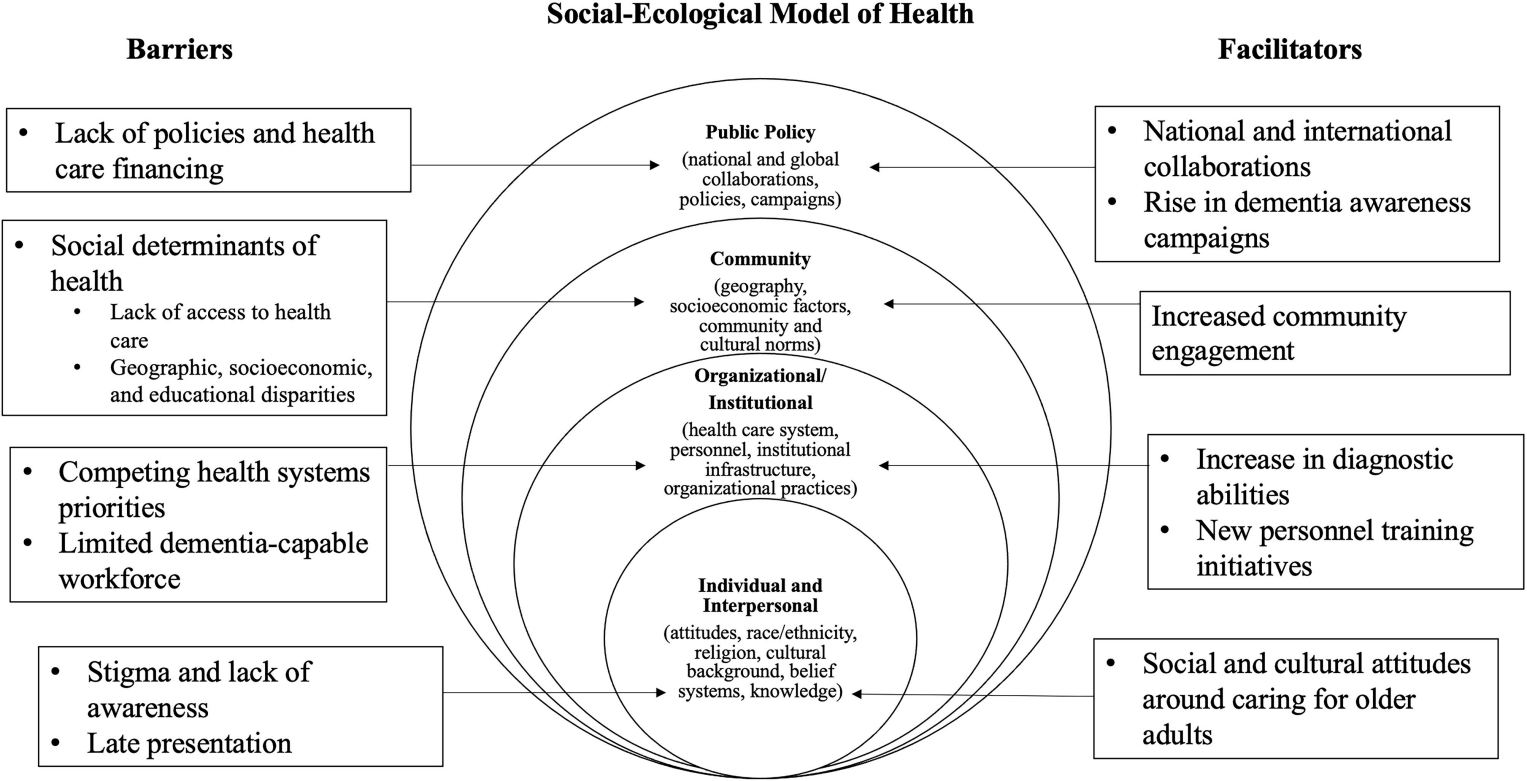
Social ecological model of health.

Respondents identified key barriers and facilitators to dementia assessment and care (Figure 1, Tables 3 and 4). We present these by Social-Ecological (SE) factor. For each barrier and facilitator we provide a description of the theme and one or two exemplary quotations in the text. Additional quotations from a range of participants are presented in the tables. We found more agreement among experts around barriers to dementia diagnosis than we did around facilitators, but indicate in the text when a topic raised was a major theme endorsed by many participants or a minor theme endorsed by fewer participants.

**Table 3:** Barriers to dementia assessment and care.

**Theme by social-ecological domain**	**Description**	**Exemplary quotes**
**Individual and interpersonal factors**		
**Stigma and lack of awareness and knowledge about dementia in the population and among healthcare providers**	Dementia being seen as normal aging, denial of the disease, ageism, stigma around dementia	• ***Geriatrician, Africa:** I think the most important issue relating to the stigma in the Arabic word for dementia. This is a major barrier. The Arabic word for dementia is kharaf, okay? Kharaf means losing your mind. So still, physicians and some of the official translating groups, they use the word kharaf, which is like insane*. • ***Psychiatrist, Central America:** I believe that we need education for both the general population and health personnel. I believe that these are the two main barriers and that education would be the way to address this lack of awareness of the probem both in the population and in health personnel*. • ***Neurologist, Middle East:** People are in denial. They resist being sick. It's part of the culture. They deny, they resist it with all their power until everything falls down*.
**Late presentation**	Underdiagnosis, misdiagnosis, delayed diagnosis	• ***Neurologist, Asia:** The problem is [they] come when they are in the late onset…the family just thinks it's normal for the elderly to forget. But, if the patient has a behavioral problem, it's the problem for the family…So, when come to the doctor, usually, you know, the patient hopes that the doctor will give them one pill to fix everything, you know, a potion*. • ***Neurologist, Middle East:** I see that the family takes their family member late to the doctor…They deny that they have any problems*. • ***Neurologist, Africa:** Our health-seeking behaviors for most things, and especially things that have to do with cognition, we wait until the problem is a glaring one before we think, Oh, maybe I should go see the doctor. So, it is coming to the hospital late. It's even by, again, I mentioned this earlier, the health professional identifying the problem late, when it's already full-blown, and then thinking, Oh, maybe I should refer now*.
**Organizational/institutional factors**		
**Competing health systems priorities**	Communicable diseases, maternal health, infant health, hunger, poverty	• ***Neurologist, Africa:** The healthcare system is not set up in a way to take into account these kinds of non-communicable diseases. We are still too basic. We are still in the communicable disease phase. That's where all our focus, public health and so on, it is based. We are moving slightly toward non-communicable diseases, but it's still extremely basic. So hypertension, diabetes, heart disease. Diseases of the nervous system are very, very low down on the priority list of the government, the policy makers, and so on.?*? • ***Psychiatrist, Asia:** Psychiatric and mental health areas are financed in this country based on the leftover principle. In other words, if there is some money left in the healthcare budget, it would rather be spent on gynecology, obstetrics, or cardiology services…Recently, our [[Minister of Health]] even expressed a wish to release patients with psychiatric disorders from acute wards to free up beds for observation of patients with infectious diseases, for example*. • ***Neurologist, India:** Dementia affects the geriatric group. Now we are grappling with infections and we are grappling with so many other treatable problems that probably dementia would be marginalized when it comes to healthcare*.
**Limited dementia capable workforce**	Lack of providers, lack of training (particularly for general practitioners/primary care), lack of focus on dementia in the healthcare workforce	• ***Neuropsychologist, Africa:** So, it there's one neurologist who's based in the north of the country, another who's based in the south of the country, and then myself who's the only neuropsychologist. So, there's really a lack of personnel and human resources. There's a lack of training. For example, even nurses, you know, they have very little training about how to identify dementia, how to treat dementia or anything like that* • ***Neuropsychologist, Europe:** Across [[European country]] I do not think there are well-trained professionals…there are no professionals – neuropsychologists in [these] places unfortunately. They are not aware of these techniques. It's something unknown to them*. • ***Neurologist, South America:** Psychiatrists in [[South American country]] have no training, they only assess behavioral symptoms*.
**Community factors**		
**Social determinants of health** Lack of access or differential access to healthcare • Geographic, socioeconomic, and educational disparities	Access to healthcare in general, specialty care, or affordable care Geographic, socioeconomic, education, literacy, culture, language	• ***Neurologist, Middle East:** The general neurologist, there were like maybe two in the government hospital, two or three total more than 5 years ago. Maybe two or three at most. And you have about five or six million people population, with absence] of access to the government hospital. So there was no way they can treat these patients. There is no way. Maybe the one who has certain connections, the one that has powerful friends, there is no way you can treat that whole amount of patients*
		• ***Neurologist, Europe:** It will all depend on the resources that the referral center that you were assigned to has… If you are lucky you will have access to a center that has a memory clinic, and I insist, the majority of the patients do not get this type of attention. Regarding memory clinics, there are a lot of inequities regarding neuropsychological testing and biomarker access*.
**Public policy**		
**Lack of policies and healthcare financing emphasizing dementia**	Financing, policies	• ***Geriatrician, Latin America:** Another barrier is the economical one, and there is where we come into the game. As we know, this is a disease for which the treatment is extremely expensive. At least here in [[country]] the cost can be around $150 to $300 dollars per month if patches or any type of drug for Alzheimer is used*. • ***Neurologist, Africa:** Is there a national policy to try and help with dementia? I do not think we have reached that point yet*. • ***Neurologist, Europe:** A major reason for these inequities is that the capacity of getting funding that each center has is tremendously unequal*. • *Geriatrician, Africa: MSPKR: Financing research for elderly health care like dementia is still, unfortunately, not of the priorities of the finance of research…I think it should be part of the local and international communities to put dementia care at the priorities of the financing*.

**Table 4:** Facilitators to dementia assessment and care.

**Theme by social-ecological domain**	**Description**	**Exemplary Quotes**
**Individual and interpersonal factors**		
Social and cultural attitudes around caring for older adults	Cultural infrastructure around caring for older adults; Caregiver/family/patient support available for people with dementia/older adults	• ***Neurologist, Asia:** So I think in most areas, [the] care model is a bit like as a family unit, meaning that – one thing to note that in Taiwan, almost 90 percent of our patients are never institutionalized during the dementia disease process. It's a very stigmatic thing regarded as the son and daughter failed the parents…in a more rural area, they might take care as a village…the whole village, all the elders that are healthy would take care of the elders that are less healthy, or the village would take care of them*. *So you do not own a particular elder – you own every elder*. • ***Psychiatrist, Africa:** What I can say is working well is care because of the sculpture of our family system. You know, we tend to care for our own, so we tend to do – you know, if your grandmother has dementia or your great aunt has dementia, chances are that they will never go without care because we care for our own at home. So, I think that's the good thing*.
**Organizational/institutional factors**		
Increase in diagnostic abilities	Increase in specialists, more testing available, more technology available (but mostly focused on specialty centers)	• ***Geriatrician, Asia:** 20 years ago, physicians who went oversea came back to adapt and validate various cognitive tests in [Asian country] versions. Most work were confined in specialized fields [like neurology]. Even among neurologists or psychiatrists, there were a few who took interest in dementia. Ten years ago, people grew more interested in dementia. There have been more tests and research which combine clinical and biomarkers such as CSF biomarker, PET Scan. The past 5 years, I feel that cognitive assessment moved to a larger circle of medicine. It is talked about and put in the undergraduate curriculum. Family physicians talk about it. Other disciplines also cares more about it. [The ministry] supports public screen program and referral track. Systems are linked and have become more supportive*. • ***Neurologist, Middle East:** The number of physician neurologists were less than 10 five years ago. Now we are about 60, so that's a big improvement. Now, patients are getting much more accessibility to neurologists. They did not have that luxury before*. • ***Neurogeriatrician, South America:** In these last 5 years, more trained neuropsychology professionals have arrived. Now there is more knowledge of the tests*.
New personnel training initiatives	Increased initiatives to train more clinicians in dementia-specific topics	• ***Geriatrician, Asia:** 20 years ago…most work [was] confined in specialized field [like neurology]. Even among neurologists or psychiatrists, there were a few who took interest in dementia. 10 years ago, people grew more interest in dementia. There have been more tests and research…the past 5 years, I feel the cognitive assessment move to a larger circle of medicine. It is talked about and put in undergraduate curriculum. Family physicians talk about it. Other disciplines also care more about it. [The ministry] supports public screen program and referral track. Systems are linked and become more supportive.?* • ***Neurologist, Latin America:** In the past there were not even dementia classes in the medical course, this appeared later, I was one of the initiators, today the geriatrics has a course in dementia, psychiatry has a course in dementia and in universities there is a course in dementia. We have a course on dementia at congresses. It increases visibility. This occurs at our university and also at other universities. [This university] is a knowledge generating center, and this is spreading and all doctors end up knowing more dementia. And this is very important*.
**Community factors**		
Increased community engagement	Community engagement or dissemination of knowledge about dementia	• ***Neurologist, Asia:** At least the few areas that I work at, they can approach the chief of the community center. So you usually have a chief, like a village chief…there would be this person that's like responsible for the community, and then organize events, like be responsible for the local community center. So question, and you ask them, right - okay, so who is 65 and above in your community, and who do you think has a concern of a cognitive kind?*
**Public policy factors**		
Rise in dementia awareness and awareness campaigns	Media, change in awareness, increased community engagement	• ***Neurologist, Africa:** I think there has been an attempt from our side, also, as neurologists, to try and help people to understand this condition, and I think that has helped medical students, newly qualified doctors, residents in training, and physicians to be more aware of this diagnosis*.
National and international collaborations	Coordinated policy efforts and involvement in international research initiatives, collaborations, research support, international training, and new initiatives/new policies	• ***Psychiatrist, Latin America:** We collaborate with the national Alzheimer's Association and the national Alzheimer's Federation. We participated in many of their activities directed toward family members and the general public. Also, in radio and television campaigns directed at to general public, for example, in September*.

### Barriers

#### Individual and Interpersonal Factors

##### Theme 1: Stigma and Lack of Awareness and Knowledge About Dementia

Every respondent reported major challenges related to either stigma or a lack of knowledge and awareness about dementia in the general population. Some of the issues related to awareness that they identified included seeing dementia as part of normal aging, denial of the disease, and ageism. Many felt that more education would remedy this lack of awareness. For a few participants, stigma emerged in relationship to cultural beliefs about cognitive impairment, such as beliefs about symptoms of dementia being connected to witchcraft. The issue of stigma and awareness was exemplified by a geriatrician from Latin America who discussed the relationship between stigma and discrimination,

*Geriatrician, Latin America: One of the main barriers is that there is stigma in this country. When families find out that their loved one is sick, they do not make it public…discrimination toward people with brain diseases is a serious cultural problem*.

Furthermore, while most experts referred to stigma when talking about the general population, representing a major theme, some also noted that there was a lack of awareness about dementia among clinicians, as well, particularly general practitioners. For those that identified this theme among clinicians, the sense of stigma and lack of awareness involved clinicians avoiding fields that focus on dementia, lack of expertise, lack of time or ability to gain expertise, or disinterest. Those that identified this issue related to awareness felt that lack of awareness created barriers to good dementia care.

A geriatrician in Africa articulated this challenge of lack of awareness among healthcare providers.

*Geriatrician, Africa: Healthcare providers…are still not interested to know how to diagnose. They are interested to know about [dementia]. They think it is the hot topic they cannot touch. We need to remove this stigma from the healthcare providers themselves*.

##### Theme 2: Late Presentation

Another prominent theme identified in the majority of interviews was the issue of late presentation, where patients show up at the doctor when they already have severe dementia and when it is more challenging to address their needs. In some interviews, late presentation was connected to lack of awareness, while in others, the experts discussed issues such as access to care or the lack of expertise among general practitioners to diagnose dementia until later stages in the disease. In an example where late presentation was connected to a lack of awareness, a neurologist in our study explained.

*Neurologist, Africa: By the time patients present, it's rather late…by the time people come, they are often moderately advanced to advanced. There are some problems with that because people have lost the ability now to, say, for example, execute a will*.

#### Organizational and Institutional Factors

##### Theme 3: Competing Health System Priorities

At the organizational and institutional levels, nearly all experts noted the challenge of competing health systems priorities. Some examples provided of competing priorities included the health of younger people or pregnant women. Others suggested that dementia was not a priority because of a focus on infectious and other treatable diseases. In these discussions of competing priorities, many participants noted that these other health issues often take precedent over the aging population, which some explained shaped decisions about resource allocation. Two experts in our study exemplified these challenges:

*Psychiatrist, Latin America: In our country these needs are not a priority because there are the needs of children, of pregnant women. There is still no clear recognition of all the emergent needs that we are facing with the population's aging…there are other problems that are a priority, like extreme poverty. When people are worried about what will they have to eat that day, or what their children are going to eat, they do not prioritize memory issues*.

*Neurologist, Asia: We are grappling with infections and we are grappling with so many other treatable problems that probably dementia would be marginalized when it comes to healthcare*.

##### Theme 4: Limited Dementia Capable Workforce

Many of the challenges raised by participants in our study centered around the healthcare workforce. Respondents from every region noted limited personnel and human resources required to address dementia diagnosis and care, including lack of neuropsychologists and neurologists. Some articulated this issue by providing an estimate of number of neurologists available across the entire population. A psychiatrist articulated a theme that was echoed by many of the experts in our study,

*Psychiatrist, Asia: There are practically no specialists who work in this area….there is no prestige in working with dementia, there are no gerontologists in the whole country…in [[largest medical school in the country]] the topic of dementia gets 2 h of teaching*.

Most participants also noted a need for more training focused on dementia, especially among general practitioners. Some respondents felt that working in dementia was not a popular choice for clinicians in their setting, which is related to the issues above regarding awareness and stigma among healthcare personnel, while another expert related this to concerns about lack of prestige for those who choose to work with the aging population.

#### Community Factors

##### Theme 5: Social Determinants of Health

At the community level, barriers identified fell within a framework of social determinants of health, such as lack of access to healthcare and geographic, socioeconomic and educational disparities. Access issues most participants raised included both lack of access and differential access to dementia-specific healthcare. Reasons that emerged across interviews included cost and lack of specialists, as noted above. Nearly every expert reported geographic differences between rural and urban areas, or differential access based on public vs. private healthcare systems. A neurologist illustrated this issue of both urban and rural differences, as well as access to specialty hospitals.

*Neurologist, Middle East: Half of the country is in the cities, and the other half are living like [[Bedouins]] and all that, so they're living in the rural areas. They have access to primary care, but not more than that, not secondary or tertiary hospitals.?*?

Related to dementia specialty care, some experts identified a lack of access to key tools needed to do a diagnostic workup. These included lack of access to neuroimaging, cognitive assessment tools, imaging, and biomarker testing, with most experts reporting a lack of standardized approach to dementia care.

*Neurologist, Europe: If you are lucky you will have access to a center that has a memory clinic. The majority of patients do not get this type of attention*.

Others noted disparities due to socioeconomic, and educational factors, which included inadequate testing and validation in patients' languages and cultures. For example,

*Neuropsychologist, Latin America: For the population that has very low socioeconomic conditions...accessing a specialized diagnosis, like the one we do here in our fourth level hospital, is very difficult. They do not come easily, almost never…the way we diagnose, it does not include illiterate patients. Those with low education do not come to our center*.

#### Public Policy Factors

##### Theme 5: Lack of Policies and Healthcare Financing Emphasizing Dementia

Finally, at the public policy level, most experts identified lack of policy and healthcare financing as significant barriers, particularly regarding implementing new plans. Some experts discussed the lack of financing for training in specialties related to dementia, while others focused on the cost of care or the lack of financing for research related to dementia. In some cases, policies and plans existed on paper with no plan for implementation, as exemplified by a neurologist:

*Neurologist, Africa: We have a National Dementia Plan, but, for its implementation, resources and stakeholder support is needed. We need bigger and more global efforts*.

### Facilitators

#### Individual and Interpersonal Factors

##### Theme 1: Social and Cultural Infrastructure Around Caring for Older Adults and Those With Dementia

Some participants noted strengths of the social and cultural systems in their countries, for example, respect for the elderly, or availability of caregivers and family networks to support people with dementia, though these strengths were not identified by the majority of participants. In some cases they connected the lack of a public care infrastructure with the need for families to be so involved, while in others they spoke of the culture of caring for older adults. A neurologist explained the role of the family structure as a strength in dementia care,

*Neurologist, Asia: One thing which is very, very strong in our sector is that our family structure helps us in the management post-diagnosis...we don't have dementia care centers...our dementia care system is the center of the patient's home*.

#### Organizational and Institutional Factors

##### Theme 2: Increase in Diagnostic Abilities

There was wide variation across sites in what is included in the diagnostic workup, which has been explored in a previous paper by our group (16). For a few experts, this involved adopting new guidelines and standardized protocols for diagnostic assessment. Others referred directly to new diagnostic tests in use, the availability of neuropsychological testing, and new technology available such as MRI machines or biomarker testing. One neurologist exemplified these changes,

*Neurologist, Europe: Now we have at the hospital a Lumipulse machine and the cerebrospinal fluid biomarker determination is…routinely tested at the hospital. Before, the cost of this biomarkers had to be covered by research. Now, they are covered by the national health care system*.

##### Theme 3: New Personnel Training Initiatives

Related to the above theme, in many of the discussions about the increase in diagnostic abilities, the experts connected these abilities to the more recent increase in personnel able to conduct diagnostic assessments, such as neuropsychologists. In some settings, new training programs were developed to build capacity. Some noted an increase in providers able to do dementia diagnosis over the last 5–10 years. Many of these initiatives were enabling better use of cognitive tests and increased training in dementia.

*Neurologist, Latin America: The fact is that in the past there were not even dementia classes in the medical course… today geriatrics has a course in dementia, psychiatry has a course in dementia and in universities there is a course in dementia...It increases visibility…this I think is positive…it is giving more importance to the theme*.

#### Community Factors

##### Theme 4: Increased Community Engagement

Some interview participants noted an increase in community engagement around caring for the elderly and specifically around those with cognitive impairment. Those that articulated this theme shared ways in which members of the community were getting involved to increase community engagement, for example, by participating in creating a dementia care plan, increasing awareness about dementia through neighborhood participation, or becoming involved in implementing assessments. A neurologist explained,

*Neurologist, Asia: We are trying to include more people that are in the community into the dementia care plan. So, once this chief in the village should be included when we try to discuss policy to try to increase dementia awareness*.

A geriatrician in our study explained the way former caregivers of people with Alzheimer's disease were getting involved in his setting by learning by volunteering and learning how to implement cognitive tests:

*Geriatrician, Latin America: We also have other kind of volunteers that are not professionals. They have been caregivers of patients with Alzheimer. We have a very solid group of people whose parents passed away and are now volunteering to teach other caregivers how to take care of patients. At the same time, they are receiving training on how to detect Alzheimer. We have provided them all with a training that I call ABC. This is basic training in which they learn to administer the Minimental or the mini cognitive tests. They can administer them in their neighborhoods, in their families or in their communities. When we make the detection campaigns, they come with us*.

#### Public Policy Factors

##### Theme 5: Rise in Dementia Awareness and Awareness Campaigns

Most respondents noted that there have been changes in awareness over the last 10 years even amidst an atmosphere of stigma around the disease. For example, many participants reported an increase in awareness campaigns and media initiatives. A few reported the increase in awareness among healthcare personnel, for example, primary care physicians who were more aware of when to refer patients, or the development of memory care sites that could accept referrals.

*Geriatrician, Latin America: Media is covering this topic. Five years ago, they did not. I think that within the millennium development objectives, the benefit that has been obtained is that the [country's] population is more aware about the disease. People know a little more about alarm symptoms and would be willing to [go] to a center that offers them help*.

Others noted the increase in awareness at the population level, which is connected to the theme discussed above about the increase in community engagement.

*Psychiatrist, Asia: Normalization used to be the case in the past. Like, in the 90s, everyone used to think that forgetfulness is a part of normal aging, it is expected. But now, particularly in [[region of country]], there are increasing social efforts about awareness of dementia, with the help of primary care physicians, psychiatrists, and neurologists; so other medical professionals are largely aware of what dementia is*.

##### Theme 6: National and International Collaborations

Some experts in our study suggested that there has been an increase in both national and international collaborations around dementia. While these collaboration took different forms depending on the country, some examples that participants provided included coordinated policy efforts, involvement in international research initiatives, international training, and new policy initiatives. Some examples of the approaches to building collaborations both nationally or internationally included the creation of national dementia plans, collaborations with associations or NGOs, international research consortiums, and national health surveys.

## Discussion

In this study, we identified facilitators and barriers to dementia assessment and care based on the experiences of dementia experts in global health settings. Prior global health studies in dementia have focused on the epidemiology of dementia (1), emphasizing identifying disease frequencies across regions, and have looked at risk factors such as education, socioeconomic status, and access to healthcare (17). Other work has focused on cognitive testing, and its cultural or linguistic applicability (16, 18). Our findings are confirmatory of many issues raised in prior studies, including the 2021 World Alzheimer's Report (19). These include challenges such as limited access to healthcare resources, including lack of clinicians, the limited primary care infrastructure, and the need for more clinicians skilled in dementia diagnosis; challenges doing diagnostic testing in populations with low education; cultural factors; the role of comorbid conditions; and costs of doing dementia diagnosis. However, we also note that our qualitative approach to interviewing dementia experts in the global health context is unique, and using their voices to illustrate previously-identified issues provide more nuance and gives voice to the on-the-ground perspectives. Furthermore, while other studies have looked primarily at challenges and barriers, we also present facilitators and strengths that emerged in our interviews, though others have noted similar opportunities as we found, such as improving medical education and the need to build more public awareness. Our study used a qualitative approach to gain the global perspective of experts who are on-the-ground in health systems.

In 2013, the G8 summit committed to focus on dementia as a global health priority, and emphasized the importance of a multi-sectorial approach to addressing dementia, particularly in LMICs. Our findings identify challenges that cross multiple sectors, while strengths identified indicate a multi-sectorial approach to addressing the challenge of dementia. We identified the following key areas based on our results: (1) raising dementia awareness while building on social and cultural strengths; (2) building dementia care workforce capacity across the health system, including generalists, given disparities in access to specialty care; and (3) building and strengthening international collaborations to support the integration of new tools, policies, and approaches to address gaps in dementia assessment and care.

### Raising Dementia Awareness While Building on Social and Cultural Strengths

Awareness (17, 20, 21), knowledge (22), and stigma are commonly cited barriers to dementia prevention, treatment, and care in the global health context (17, 23, 24). Findings from our study also reinforced these challenges. We similarly identified the need for more dementia awareness to address issues around stigma or seeing dementia as part of normal aging (21) that may result in late presentation, as many research participants suggested. However, we also identified social and cultural strengths, such as approaches to caring for older adults and ways that cultural leaders have been engaged, that may provide a foundation for more effective community-based work building dementia awareness. Supporting this approach, previous work in both epidemiological and clinical research has emphasized the importance of recognizing diversity in sociocultural factors across cultures and societies, and how these shape people's understanding of dementia and prevention and care management interventions (20). Tailored interventions that center the experiences and voices of local communities as well as geopolitical and social contexts are needed to develop awareness and produce a more targeted response in LMICs (9, 25).

Furthermore, we found that the need for dementia awareness extended to generalist healthcare providers. Focusing awareness campaigns on non-expert healthcare personnel is an important next step for improving dementia care globally. However, we also found that experts *did* identify improvements in dementia awareness over the last 10 years, offering examples of new educational campaigns, media approaches, and community outreach that set the stage for building more widespread understanding of dementia.

### Building Dementia Care Workforce Capacity Across the Health System, Including Generalists, Given Disparities in Access to Care

In our study, most participants identified the need for a more robust dementia care workforce and reported challenges that exist around lack of training and capacity to diagnose and care for people with dementia. Although much of the literature on the dementia care workforce focuses on HICs (26), recent work has discussed the need for “task shifting” in the global health setting- the concept of moving dementia-related tasks from expert health providers to health workers with less training, such as community health workers (27, 28). These types of workforce solutions can help to address the need for dementia capable providers (13). Furthermore, much work has emphasized the role of primary care providers in dementia care in HICs, including the barriers faced as well as attitudes about dementia care (14, 15, 29, 30). Given the disparities we identified in access to specialty care, building dementia care capacity among generalist clinicians may improve dementia care more broadly in LMICs. Emphasis is needed on incentives to bring generalists the education and training they need to do dementia-capable work with patients (31). Finally, challenges around training go hand-in-hand with the need for feasible dementia assessment tools (16). Work is needed to develop the healthcare infrastructure related to dementia assessment tools that can be easily used and interpreted by generalists (16).

*Building and strengthening international collaborations to support the integration of new approaches, tools, and policies to improve dementia assessment and care globally*.

Experts in our study noted deficiencies in national policies around dementia. However, many also identified the development of international collaborations as a strength that can be built on. International collaborations and global coordination is important both for inclusiveness in research to ensure representative research cohorts, as well as to address the unequal impact of dementia across the globe (32). A key example is Alzheimer's Disease International's coordination of the development of national dementia care plans and writing annual reports about dementia in LMIC. Other international consortiums are also working to set research priorities, improve diagnosis and care, and both address and record global challenges related to dementia care (2).

### Limitations

This study has several limitations. (1) We were unable to sample from all LMICs meeting the inclusion criteria, though we had regional representation. (2) We relied on interviews with one to two neurology experts from each country sampled, and therefore cannot generalize these results. More work and a larger sample are needed. (3) Participants in our study were not asked specifically about the different levels of the social ecological model, as the model was used as a way to organize inductive findings of our exploratory study. It is possible the experts would have identified additional barriers an facilitators if the interview had been conducted in a systematic way using this model. (4) We limited our interview to dementia experts, though we asked about challenges in non-expert settings. Future work is needed with broader representation of generalists, community health workers, and other informants in these settings to identify whether their perceptions are consistent with expert perspectives and to identify additional facilitators and barriers. (5) Finally, this study was not designed to compare and contrast differences across countries, and particularly did not look at differences between MIC and LICs. Future work would sample from more LICs and conduct a comparative analysis.

### Future Directions

Findings from this study provide insights for prioritizing dementia assessment and care capacity-building in LMICs as a global health priority. We also identified ways that tailored public health approaches can strengthen dementia assessment and care at the individual, community, national, and multi-national levels.

## Data Availability Statement

The raw qualitative data is not available publicly due to the need to protect the privacy of interviewees, who did not consent to have transcripts of their interviews shared. However, the authors may make summary data available on a case-by-case basis as allowed by human subjects policies. Requests to access the data should be directed to the corresponding authors.

## Ethics Statement

The studies involving human participants were reviewed and approved by the University of California San Francisco. Written informed consent from the participants was not required to participate in this study in accordance with the national legislation and the institutional requirements.

## Author Contributions

ABS designed and conceptualized study, analyzed and interpreted the data, drafted and revised the manuscript for intellectual content, obtained funding, and major role in the acquisition of data. TA-R designed and conceptualized study, analyzed and interpreted the data, and revised the manuscript for intellectual content. ET, SP, MP-C, SK, LM-M, MO, and MD designed and conceptualized study, major role in the acquisition of data, revised the manuscript for intellectual content. SZ, AA, and HI had major role in the acquisition of data and revised the manuscript for intellectual content. CG had major role in acquisition of data and manuscript preparation. BM revised the manuscript for intellectual content. VV designed and conceptualized study and revised the manuscript for intellectual content. KP designed and conceptualized study, obtained funding, and revised the manuscript for intellectual content. All authors contributed to the article and approved the submitted version.

## Funding

This study was supported by the Global Brain Health Institute, the UCSF Population Health & Health Equity Scholars Program, the National Institute on Aging (K01AG059840-01A1), the National Heart, Lung, and Blood Institute (K23HL148530), and the National Institute of Neurological Disorders and Stroke (UG3NS105557-01).

## Conflict of Interest

The authors declare that the research was conducted in the absence of any commercial or financial relationships that could be construed as a potential conflict of interest.

## Publisher's Note

All claims expressed in this article are solely those of the authors and do not necessarily represent those of their affiliated organizations, or those of the publisher, the editors and the reviewers. Any product that may be evaluated in this article, or claim that may be made by its manufacturer, is not guaranteed or endorsed by the publisher.
