# Supplementary material for: Facilitators and Barriers to Dementia Assessment and Diagnosis: Perspectives From Dementia Experts Within a Global Health Context
Source: Front Neurol. 2022 Mar 28;13:769360. doi: 10.3389/fneur.2022.769360 (PMC8997042; doi:10.3389/fneur.2022.769360)
Supplement: Supplementary file 1 [file Data_Sheet_1.docx]

**Appendices**

Appendix 1: Consolidated criteria for reporting qualitative studies (COREQ) 32-item checklist

Appendix 2: Pre-interview survey

Appendix 3: Semi-structured interview guide

**1. Supplementary Table 1: Consolidated criteria for reporting qualitative studies (COREQ) 32-item checklist**

| **No. Item** | **Guide questions/description** | **Reported on Page #** |
| --- | --- | --- |
| **Domain 1: Research team and reﬂexivity** | | |
| ***Personal Characteristics*** | | |
| 1. Interviewer/facilitator | Which author/s conducted the interview or focus group? | *Methods, page 5* |
| 2. Credentials | What were the researcher’s credentials? e.g., PhD, MD | *Author list, page 1* |
| 3. Occupation | What was their occupation at the time of the study? | *n/a* |
| 4. Gender | Was the researcher male or female? | *n/a* |
| 5. Experience and training | What experience or training did the researcher have? | *Methods, page 5* |
| ***Relationship with participants*** | | |
| 6. Relationship established | Was a relationship established prior to study commencement? | *Methods, page 5* |
| 7. Participant knowledge of the interviewer | What did the participants know about the researcher? e.g., personal goals, reasons for doing the research | *Research purposes*  *were described in invitation emails and interview introductions* |
| 8. Interviewer characteristics | What characteristics were reported about the interviewer/facilitator? e.g., bias, assumptions, reasons and interests in the research topic | *Interviewers briefly introduced themselves during the interview and shared research objectives with participants* |
| **Domain 2: Study design** | | |
| ***Theoretical framework*** | | |
| 9. Methodological orientation and Theory | What methodological orientation was stated to underpin the study? e.g., grounded theory, discourse analysis, ethnography, phenomenology, content analysis | *Methods, pages 5* |
| ***Participant selection*** | | |
| 10. Sampling | How were participants selected? e.g., purposive, convenience, consecutive, snowball | *Methods, page 5* |
| 11. Method of approach | How were participants approached? e.g., face-to-face, telephone, mail, email | *Methods, page 5* |
| 12. Sample size | How many participants were in the study? | *Results, page 5* |
| 13. Non-participation | How many people refused to participate or dropped out? Reasons? | *Methods, page 5* |
| ***Setting*** | | |
| 14. Setting of data collection | Where was the data collected? e.g., home, clinic, workplace | *Methods, page 5* |
| 15. Presence of non-participants | Was anyone else present besides the participants and researchers? | *No, the interviews were conducted on a 1-on-1 basis* |
| 16. Description of sample | What are the important characteristics of the sample? e.g., demographic data, date | *Results, page 5; Tables 1 & 2* |
| *Data collection* | | |
| 17. Interview guide | Were questions, prompts, guides provided by the authors? Was it pilot tested? | *Methods, page 5*  *Supplementary Table 3* |
| 18. Repeat interviews | Were repeat interviews carried out? If yes, how many? | *No* |
| 19. Audio/visual recording | Did the research use audio or visual recording to collect the data? | *Methods, page 5* |
| 20. Field notes | Were ﬁeld notes made during and/or after the interview or focus group? | *No* |
| 21. Duration | What was the duration of the interviews or focus group? | *Methods, page 5* |
| 22. Data saturation | Was data saturation discussed? | *Methods, page 5* |
| 23. Transcripts returned | Were transcripts returned to participants for comment and/or correction? | *No* |
| **Domain 3: Analysis and ﬁndings** | | |
| ***Data analysis*** | | |
| 24. Number of data coders | How many data coders coded the data? | *Methods, page 5* |
| 25. Description of the coding tree | Did authors provide a description of the coding tree? | *Tables 3 and 4* |
| 26. Derivation of themes | Were themes identiﬁed in advance or derived from the data? | *Methods, pages 5* |
| 27. Software | What software, if applicable, was used to manage the data? | *Methods, page 5* |
| 28. Participant checking | Did participants provide feedback on the ﬁndings? | *N/A (methodology not used)* |
| ***Reporting*** | | |
| 29. Quotations presented | Were participant quotations presented to illustrate the themes/ﬁndings? Was each quotation identiﬁed? e.g., participant number | *Results, throughout*  *Tables 3 & 4* |
| 30. Data and ﬁndings consistent | Was there consistency between the data presented and the ﬁndings? | *Results, throughout*  *Tables 3 & 4* |
| 31. Clarity of major themes | Were major themes clearly presented in the ﬁndings? | *Results, throughout, Tables 3 & 4* |
| 32. Clarity of minor themes | Is there a description of diverse cases or discussion of minor themes? | *Results, throughout, Tables 3 & 4* |

**2. Supplementary Table 2: Pre-interview survey**

| **Question** | **Response options** |
| --- | --- |
| What is your gender? | - Female - Male - Other |
| What is your race? (Please select all that apply) | - White - Black or African American - American Indian or Alaska Native - Asian - Native Hawaiian or Pacific Islander - Other, please enter [free text] |
| Are you of Hispanic or Latino decent? | - Yes - No |
| What is your primary specialty? | - Neurology - Geriatric Medicine - Internal Medicine - Psychiatry - Neuropsychology - Nursing - Other, please specify [free text] |
| Please estimate the percentage of your time you dedicate for each (should total to 100%) | - Patient care (%) - Research (%) - Teaching and Mentoring (%) - Administration (%) - Other (%) please specify [free text] |
| How many hours do you work per week? | [free text] |
| How many hours per week do you spend on dementia diagnosis (in patient care, research, or teaching and mentoring)? | [free text] |
| How many years have you been in clinical practice? | [free text] |
| How many of those years have you been involved in dementia diagnosis in clinical practice? | [free text] |
| How would you describe your primary practice affiliation? Please select all that apply | - Teaching Hospital - Public Institution - Private Institution - Research Institution - Other (please specify) [free text] |
| What providers contribute to the diagnosis of dementia in your primary clinic? Please select all that apply | - Neurologist - Psychiatrist - Neuropsychologist - Geriatrician - Internist - Primary Care Physician - Nurse - Social Worker - Speech Therapist - Other (please specify) [free text] |
| Is there a standardized institutional/national guideline for dementia diagnosis available in your center and in the country? | - Yes (please enter the name of the guideline) [free text] - No |
| On average, how many patients do you see or supervise per month? | [free text] |
| Of these patients you see or supervise each month, how many are over the age of 55? | [free text] |
| On average, how many patients do you newly diagnose with mild cognitive impairment/disorder or dementia, per month? | [free text] |
| Of these patients you newly diagnose with mild cognitive impairment/disorder or dementia per month, how many are over the age of 55? | [free text] |
| Please estimate the percentage of newly diagnosed patients in your practice that receive the following as a part of their diagnostic evaluation? | - Comprehensive standardized cognitive assessment (typically involving multiple tests and taking 1 hour or more) [free text] - Brief standardized cognitive assessment (typically involving 1 test, completed in 15 minutes or less) [free text] - No standardized cognitive assessment [free text] |
| What are the most commonly used brief cognitive test(s) in your country? Please rank (1-3) the top most frequently used tools from this list | - MMSE (Mini Mental State Examination) - MoCA (Montreal Cognitive Assessment) - ADAS-Cog (Alzheimer’s Disease Assessment Scale-Cognitive Subscale) - RUDAS (Rowland Universal Dementia Assessment Scale) - Mini-Cog - GPCog (General Practitioner Assessment of Cognition) - ACE (Addenbrooke’s Cognitive Examination) - Clock Drawing Test - 7-Minute Screen - Other (please specify) [free text] |

**3. Supplementary Table 3: Semi-structured interview guide**

| **Question** |
| --- |
| Could you please describe the setting of your current clinical practice? |
| What is the process for diagnosing people with dementia in your practice? What is the process for diagnosing dementia in your country for most patients |
| What barriers to dementia diagnosis do you see in your practice? In your country? |
| What is working well with dementia diagnosis in your practice? In your country? |
| What are strengths and limitations of current standardized cognitive assessment tools in your practice? |
| What changes have you seen in cognitive assessments in your country over the last 10-20 years? |
| What changes are needed to address the growing aging population in your country? |
| Could you describe what you envision as the ideal brief cognitive assessment to address the needs in your country? |
